# Viscoat versus Visthesia during phacoemulsification cataract surgery: corneal and foveal changes

**DOI:** 10.1186/1471-2415-11-9

**Published:** 2011-04-29

**Authors:** Marilita M Moschos, Irini P Chatziralli, Theodoros N Sergentanis

**Affiliations:** 1 1st Department of Ophthalmology, University of Athens, Athens, Greece; 2 Department of Epidemiology and Biostatistics, University of Athens, Athens, Greece

## Abstract

**Background:**

Ophthalmic viscosurgical devices (OVDs) are widely used in phacoemulsification cataract surgery to maintain adequate intraocular space, stabilize ocular tissue during the operation and decrease the possible damage of the corneal endothelium. Our study has the purpose to compare the corneal and foveal changes of Viscoat and Visthesia in patients undergoing uneventful phacoemulsification cataract surgery.

**Methods:**

Participants in our study were 77 consecutive patients, who were randomized into two groups based on type of OVD used during phacoemulsification: Viscoat or Visthesia. All patients underwent a complete ophthalmological examination i.e., measurement of best corrected visual acuity (BCVA) by means of Snellen charts, intraocular pressure examination by Goldmann tonometry, slit lamp examination, fundus examination, optical coherence tomography, specular microscopy and ultrasound pachymetry preoperatively and at three time points postoperatively (day 3, 15, 28 postoperatively). The differences in baseline characteristics, as well as in outcomes between the two groups were compared by Mann-Whitney-Wilcoxon test and Student's t-test, as appropriate.

**Results:**

Intraoperatively, there was no statistically significant difference in the duration of the ultrasound application between the two groups, while Viscoat group needed more time for the operation performance. It is also worthy to mention that Visthesia group exhibited less intense pain than patients in Viscoat group. Postoperatively, there was a statistically significant difference in central corneal thickness, endothelial cell count and macular thickness between the two groups, but BCVA (logMAR) did not differ between the two groups.

**Conclusions:**

Our study suggests that Viscoat is more safe and protective for the corneal endothelium during uneventful phacoemulsification cataract surgery, while Visthesia is in superior position regarding intraoperative pain. Patients of both groups acquired excellent visual acuity postoperative. Finally, this is the first study comparing OVDs in terms of macular thickness, finding that Visthesia cause a greater increase in macular thickness postoperatively than Viscoat, although it reaches normal ranges in both groups.

## Background

Ophthalmic viscosurgical devices (OVDs) in cataract surgery were first described by Balazs et al. in 1972 [1]. Since then they are widely used in phacoemulsification cataract surgery. Specifically, OVDs maintain adequate intraocular space and stabilize ocular tissue during the operation, especially in the stages of capsulorhexis and intraocular lens (IOL) implantation [2-9]. Moreover, OVDs facilitate any surgical manoeuvres and decrease the possible damage of the corneal endothelium due to surgical trauma [2,3,6]. They are also considered to inhibit the formation of free radicals which negatively affect the corneal endothelium during phacoemulsification [2,10,11].

According to Arshinoff classification, OVDs can be divided into two groups, based on their physicochemical and rheological properties: dispersive and cohesive [12]. Dispersive OVDs have lower molecular weight, shorter molecular chains and remain in the anterior chamber more than cohesive OVDs, requiring a longer aspiration time for their complete removal. In contrast, cohesive OVDs help sustain the anterior chamber and can be easily removed due to their high cohesiveness [5,7,12]. Viscoat^®^, a combination of sodium hyaluronate 3% and chondroitin sulfate 4%, has the typical properties of a dispersive OVD, while Visthesia^® ^Intracameral, a formulation of sodium hyaluronate 1.5% and lidocaine hydrochloride 1%, demonstrates as a cohesive one [7-9]. The characteristics of the two products are shown in Table 1. It is worth mentioning that the mixture of an OVD with an anaesthetic (viscoanaesthesia) may provide more comfort to patients and makes the application of the anaesthetic easy. However, the addition of lidocaine in classic OVDs may prolong the toxic effect of lidocaine on the corneal endothelium [9,13].

**Table 1:** Physicochemical and rheological properties of Viscoat and Visthesia

Property	**Viscoat**^**^®^**^	**Visthesia**^**^®^**^
*Manufacturer*	Alcon Laboratories	Zeiss
*Ingredients*	CDS/NaHa	NaHa/LH
*Concentration (%)*	4 CDS/3 NaHa	1.5 NaHa/1 LH
*Viscosity (mPas)*	40000	500000
*Molecular weight (Dalton)*	22500 CDS/>500000 NaHa	3000000
*Osmolarity (mOsm/L)*	330	280-330
*pH value*	7-7.5	7-7.4

NaHa = sodium hyaluronate; CDS = chondroitin sulfate; LH = lidocaine hydrochloride

Under the light of the above, our study has the purpose to compare the effect of Viscoat and Visthesia on central corneal thickness, endothelial cell density, macular thickness and best corrected visual acuity (BCVA) preoperatively and at three time points postoperatively (day 3, 15, 28 postoperatively) in patients undergoing uneventful phacoemulsification cataract surgery. Also, intraoperative pain was evaluated.

## Methods

### Recruitment of patients, preoperative assessment, randomization and exclusion criteria

Participants in our study were 77 consecutive patients, who were recruited in the 1^st ^Department of Ophthalmology, University of Athens, Athens, Greece. Patients' information included age, sex and which eye would be operated. All eyes were the first to be operated, in order to avoid heterogeneity. Preoperatively, all patients underwent a complete ophthalmological examination i.e., measurement of BCVA by means of Snellen charts, intraocular pressure evaluation (IOP) by Goldmann tonometry, slit lamp examination and fundus examination. Also, optical coherence tomography (OCT) examination (Stratus OCT3, Carl Zeiss Meditec, Dublin, CA, USA) and specular microscopy (CSO SP-01 specular miscoscope) were performed by an experienced operator, to measure macular thickness and endothelial cell count (ECC) respectively. Furthermore, central corneal thickness (CCT) was measured by ultrasound pachymetry using the Ocuscan^® ^R_x_P Alcon^®^. After the preoperative examination, patients were randomized into two groups based on type of OVD used during phacoemulsification: Viscoat or Visthesia.

Exclusion criteria were corneal abnormalities, history of intraocular surgery, preoperative endothelial cell count less than 1500 cells/mm^2^, history of uveitis, diabetes, age-related macular degeneration and intraoperative complications, such as posterior capsule rupture, vitreous loss, lost nucleus, zonule dehiscence and wound leak. Moreover, one patient who developed cystoid macular edema confirmed by OCT was excluded, because he exhibited posterior capsule rupture during the operation and we included only the uneventful cases in our study.

The study was in accordance with the tenets of the Declaration of Helsinki and has been approved by the institutional review board of our hospital ("G. Gennimatas" Athens General Hospital). Written informed consent was obtained from all patients.

### Description of the procedure

On the day of surgery, the pupil was dilated with Tropicamide 0.5% (Tropixal, Demo) and Phenylephrine Hydrochloride 5% (Phenylephrine, Cooper) drops every 10 minutes for 30 minutes before surgery. All operations were performed with a standard technique by the same surgeon. The lid and the periorbital skin were cleaned and the conjunctival cul-de-sac was irrigated with povidone iodine. Proparacaine hydrochloride 0.5% drops (Alcaine^®^, Alcon laboratories) were used as topical anesthetic and were administered 10 minutes prior to the beginning of surgery. The lid and the periorbital skin were then draped and an open wide speculum was placed. The eye was irrigated with Balanced Salt Solution (BSS^®^, Alcon).

A clear corneal incision and side-port paracentesis were made. OVD, either Viscoat or Visthesia, was injected into the anterior segment and a continuous curvilinear capsulorrhexis was created with a forceps. The lens nucleus and cortex were hydrodissected with BSS. This was followed by phacoemulsification, irrigation and aspiration of cortical remnants via phaco chops methods by using Infinity™ Vision System (Alcon Laboratories). OVD infusion and implantation of the foldable posterior chamber IOL were performed using the injector system recommended for each lens. The viscoelastic material was subsequently removed and surgical wounds were hydrated with BSS. No sutures were applied. All wounds were checked for leakage and found to be watertight. The duration of the application of ultrasound during phacoemulsification and the duration of the whole operation were recorded. Immediately after the completion of surgery, patients were asked to rate their pain on a visual analog scale (VAS, range: 0-10). In specific, for VAS rating, we used a vertical line of 10 cm length. One (bottom) end of the line was marked as zero pain and the other (top) end of the line was marked as maximum pain. After instructing patients that the bottom end of the line represents no pain and severity of pain increases as you go along the line and reaches maximum pain at the top of the line, patients were asked to mark the point in the line which represented the severity of their current pain. Then distance from the bottom end of the line to the point marked by the patients was measured in millimeters and the value was taken as the pain score.

### Postoperative treatment, follow-up

All patients received the same postoperative treatment i.e., combination of tobramycin 0.3% - dexamethasone 0.1% (TobraDex^®^, Alcon) one drop four times/day, plus dorzolamide hydrochloride 2% - timolol maleate 0.5% (Cosopt^®^, Merck & Co) one drop twice/day. The topical treatment was administered for 28 days after phacoemulsification.

Three follow-up visits were scheduled for all patients: on postoperative day 3, 15 and 28. A thorough ophthalmological examination, including BCVA measurement, slit lamp examination, IOP measurement, fundoscopy, OCT scan, ECC measurement and CCT measurement, was performed in all follow-up visits by the same ophthalmologist.

### Statistical analysis

The Gaussian distribution assumption was tested using the Kolmogorov - Smirnov test. None of variables (BCVA, ECC, CCT, macular thickness, ultrasound duration, intraoperative pain) succeeded in passing the normality test, except for age and total operation duration.

The differences in baseline characteristics, as well as in outcomes between the two groups were compared by Mann-Whitney-Wilcoxon test for independent samples (referred to as MWW for reasons of brevity), as appropriate. Given that four comparisons (day 0, 3, 15, 28) took place, the Bonferroni correction for multiple comparisons was adopted; as a result the threshold of statistical significance for *p *values was set to 0.05/4 = 0.0125. Concerning BCVA, the descriptive statistics of the log of the minimum angle of resolution (logMAR) were computed as appropriate [14].

The Student t-test was used to compare age and total operation duration between the two groups, because they followed the normal distribution. Statistical analysis was performed with STATA 10.0 statistical software (StataCorp, College Station, TX, USA).

## Results

The demographic and intraoperative characteristics of the two groups are shown in Table 2. There were no statistically significant differences between groups preoperatively in age, gender, BCVA, ECC, CCT and macular thickness. Intraoperatively, there was no statistically significant difference in the duration of the ultrasound application between the two groups (p = 0.483, MWW test), while Viscoat group needed more time for the operation performance (p = 0.012, Student t-test). It is also worthy to mention that Visthesia group exhibited less intense pain than patients in Viscoat group (p < 0.0001, MWW test).

**Table 2:** Demographic characteristics and intraoperative data of the two groups

	Viscoat group (n = 41)	Visthesia group (n = 36)	p
**Continuous variables**	mean ± SD	mean ± SD	

Age (years)	77.6 ± 8.4	77.7 ± 8.7	0.949
BCVA preoperatively (LogMAR)	0.42 ± 0.22	0.41 ± 0.25	0.844
ECC preoperatively (cells/mm^2^)	2424.9 ± 449.03	2307.6 ± 382.63	0.225
CCT preoperatively (μm)	554.9 ± 43.75	560.2 ± 42.84	0.591
Macular thickness preoperatively (μm)	156.9 ± 23.33	159.2 ± 26.56	0.684
U/S duration intraoperatively (min)	0.83 ± 0.70	0.66 ± 0.38	0.483
Total operating time (min)	15.90 ± 2.97	14.43 ± 1.85	0.012
Intraoperative pain (VAS score)	3.27 ± 0.81	1.00 ± 0.76	<0.0001

**Categorical and ordinal variables**	N	N	

Sex			
*Male*/*Female*	15/26	11/25	0.577
Operated eye			
*Right/Left*	20/21	19/17	0.726

BCVA = best corrected visual acuity; ECC = endothelial cell count; CCT = central corneal thickness; U/S = ultrasound; VAS = visual analog scale

**Figure 1:**
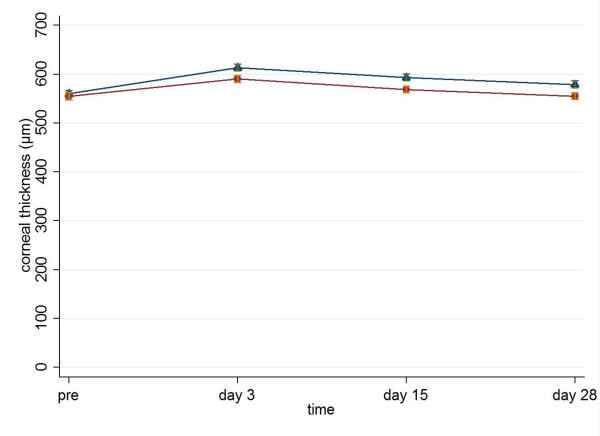
**Central corneal thickness (mean ± standard error, μm) preoperatively and on postoperative days 3, 15 and 28 in Viscoat group (dots) and Visthesia group (triangles)**.

There was a statistically significant difference in CCT between the two groups at the three time points of the follow-up (for Viscoat and Visthesia respectively: 590.5 ± 45.07 vs. 613.6 ± 41.36 on day 3, p = 0.012, MWW; 568.9 ± 44.96 vs. 593.4 ± 43.08 on day 15, p = 0.009, MWW; 555.1 ± 42.53 vs.579.3 ± 42.70 on day 28, p = 0.008, MWW, Figure 1).

**Figure 2:**
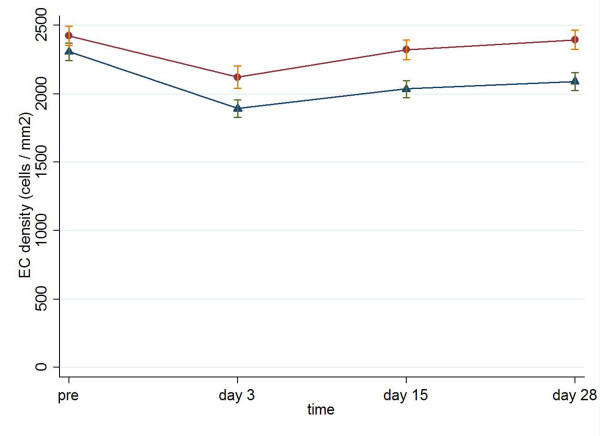
**Endothelial cell count (mean ± standard error, cells/mm**^**2**^**) preoperatively and on postoperative days 3, 15, 28 in Viscoat group (dots) and Visthesia group (triangles)**.

Respectively, there was a statistically significant difference in ECC between the two groups at the three time points of the follow-up (for Viscoat and Visthesia respectively: 2122.6 ± 526.1 vs. 1891.7 ± 385.5 on postoperative day 3, p = 0.011, MWW; 2321.9 ± 468.1 vs. 2035.3 ± 376.9 on postoperative day 15, p = 0.006, MWW; 2395.9 ± 451.2 vs. 2090.6 ± 384.7 on postoperative day 28, p = 0.003, MWW, Figure 2). Preoperative mean endothelial cell density was 2425 cells/mm^2 ^in the Viscoat group and 2308 cells/mm^2 ^in the Visthesia group (p = 0.225, MWW). Noticeably, the density decreased by 29 cells/mm^2 ^(a 1.2% loss) in the Viscoat group and by 217 cells/mm^2 ^(a 9.6% loss) in the Visthesia group 28 days postoperatively (p < 0.0001), as it is illustrated in Table 3.

**Table 3:** Endothelial cell density over time

Time period	Viscoat group	Visthesia group	p
*Preoperative ECC (cells/mm*^*2*^*)*	2424.9 ± 449.03	2307.6 ± 382.63	0.225
*Postoperative day 28 ECC (cells/mm*^*2*^*)*	2395.9 ± 451.2	2090.6 ± 384.7	0.003
*Decrease in the ECC (%)*	-1.24 ± 2.04	-9.60 ± 3.86	<0.0001

ECC = endothelial count cells

**Figure 3:**
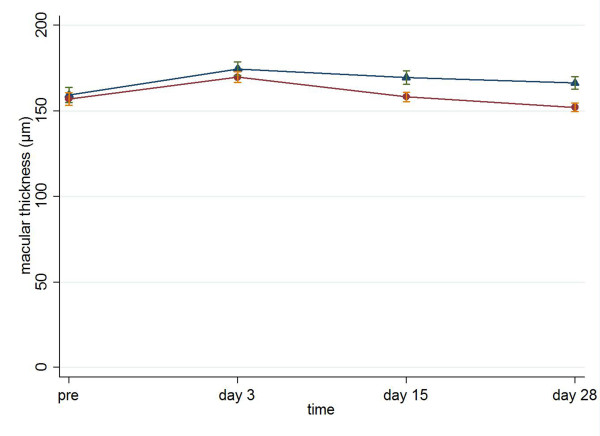
**Macular thickness (mean ± standard error, μm) preoperatively and on postoperative days 3, 15 and 28 in Viscoat group (dots) and Visthesia group (triangles)**.

Visthesia group differed significantly in comparison with Viscoat group regarding macular thickness, at the late time point of the follow-up (for Viscoat and Visthesia respectively: 169.8 ± 19.9 vs. 174.6 ± 23.5 on postoperative day 3, p = 0.330, MWW; 158.3 ± 17.7 vs. 169.5 ± 22.9 on postoperative day 15, p = 0.018, MWW; 152.0 ± 16.3 vs. 166.3 ± 21.4 on postoperative day 28, p = 0.002, MWW, Figure 3).

**Figure 4:**
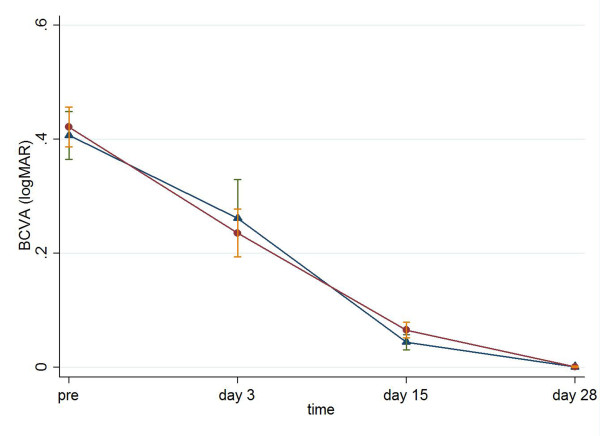
**BCVA (mean ± standard error, logMAR) preoperatively and on postoperative days 3, 15 and 28 in Viscoat group (dots) and Visthesia group (triangles)**.

BCVA (logMAR) did not differ between the two groups (for Viscoat and Visthesia respectively: 0.24 ± 0.24 vs. 0.26 ± 0.37 on postoperative day 3, p = 0.238, MWW; 0.07 ± 0.09 vs. 0.05 ± 0.08 on day postoperative day 15, p = 0.041, MWW; 0.0014 ± 0.0078 vs. 0.001 ± 0.0083 on postoperative day 28, p = 0.926, MWW, Figure 4).

Worthy of note, intraoperative pain was not associated with operation time in either groups (Spearman's rho = 0.185, p = 0.247 in the Viscoat group and Spearman's rho = 0.176, p = 0.304 in the Visthesia group).

## Discussion

The principle message of our study is that Viscoat provides better protection to corneal endothelium in comparison with Visthesia during uneventful phacoemulsification cataract surgery. This is apparent, because Visthesia caused a larger endothelial cell loss, as well as more corneal edema than Viscoat. Nevertheless, the two OVDs had no significantly difference as far as the BCVA acquired postoperatively.

The main reason for using OVDs in cataract surgery is to prevent damage of the corneal endothelium. Dispersive OVDs, such as Viscoat, are considered to protect the endothelium better than cohesive OVDs, because of their lower specific surface properties [2]. However, they need longer aspiration time to be removed from the anterior chamber and it may damage the corneal endothelium in this way [2-9,15]. In our study, there was a significant difference in the duration of the operation, noting that Viscoat group needed more time for the surgery, although the duration of the ultrasound application did not differ between the two groups. On the other hand, cohesive OVDs, like Visthesia, are more effective in keeping a deep anterior chamber than the dispersive one. They also tend to escape from the anterior chamber, leaving endothelial cells without protection [6,16]. In addition to this, there are studies showing that Visthesia could be harmful to the corneal endothelium [17,18].

The damage of the corneal endothelium can be evaluated by measuring the endothelial cell decrease after surgery [9]. Adult human corneal endothelium is considered a non-replicative tissue and there is a natural decrease in endothelial cell density by age [19]. Dispersive OVDs are expected to cause less endothelial cell loss, as they protect better the corneal endothelium. This is in line with Glasser et al. who observed less endothelial cell loss in eyes receiving Viscoat than in those receiving 1% sodium hyaluronate (Healon^® ^OVD, AMO), a cohesive OVD [20]. On the contrary, Holzer et al. suggested that 2.3% sodium hyaluronate (Healon^® ^5, OVD, AMO) had lower mean endothelial cell loss in comparison with Viscoat, while Lane et al. found similar amounts of endothelial cell loss in eyes receiving cohesive and dispersive OVDs [5,21]. In our study, there is a statistically significant difference in endothelial cell loss between the two groups. The mean endothelial cell loss was for Visthesia 9.6% and for Viscoat 1.2%. Of note, the endothelial cell decreases for several OVDs are between 0.3% and 20.32%. Our results are comparable with those in other investigations published in the literature [3,5-9,16,20-25].

Another sign of functional damage of the corneal endothelium is the CCT. Corneal thickness increases when the pump and barrier functions of the endothelium are damaged, affecting the clarity of the cornea [24,26]. If there is a certain decrease in endothelial cells and an increase in corneal thickness, corneal edema appears [6]. There are several studies reporting an acute reversible increase in CCT after phacoemulsification cataract surgery [3,15,24,27]. Kiss et al. found no significantly difference in corneal edema and endothelial cell loss, when comparing Viscoat and 2% hydroxypropyl methylcellulose (Ocucoat^®^, Bausch & Lomb) [24]. On the contrary, Koch et al. observed better endothelial protection with Viscoat vs. Healon [16], in parallel with Storr-Paulsen et al. who stated that dispersive OVDs are more protective for the corneal endothelium [6]. The latter is in accordance with our results showing that Viscoat differ significantly in CCT and endothelial cell loss in comparison with Visthesia, supporting the fact that Viscoat provides more protection to the corneal endothelium. Concerning Visthesia, in line with our results, Valimaki et al. noted that Visthesia presented an increased risk for postoperative corneal edema [13].

Furthermore, an interesting finding of our study was that macular thickness was significantly higher in Visthesia group than in Viscoat one on postoperative day 28. This is the first study examining the possible effect of OVDs on macular thickness. It is well established that foveal thickness could be increased postoperatively [28,29]. However, according to Johansson et al., intracameral lidocaine did not produce more pronounced macular edema than other methods of anesthesia [30]. In our study, there was a little increase (4%) in macular thickness in Visthesia group, while in Viscoat group macular thickness was lower postoperatively (3% decrease). Nevertheless, macular thickness of both groups was normal and cystoid macular edema was not developed, except for one patient in Visthesia group who was excluded.

Noticeably, postoperative BCVA did not differ between the two groups, although there were differences in ECC, CCT and macular thickness. A possible explanation is that the aforementioned parameters reached normal ranges postoperatively in both groups and therefore did not affect negatively BCVA. It is worthy to say that all patients presented with excellent BCVA on postoperative day 28, suggesting that uneventful phacoemulsification cataract surgery is beneficial for patients' visual acuity.

Concerning intraoperative pain, Visthesia group exhibited less intense pain than patients in Viscoat group. Combining an OVD with an anaesthetic agent is very important, as this viscoelastic concept provides comfort to both patients and surgeons during the operation [9,31]. However, lidocaine hydrochloride may be the responsible agent for the differences in ECC, CCT and macular thickness between the two groups. Nevertheless, experimental works in the rabbits showed that intracameral use of lidocaine 2% induces few ultrastructure alterations in the corneal endothelial cells [32]. Also clinical studies demonstrated that intracameral lidocaine does not induce significant macular edema than the standard regimen of topical mydriatics plus intracameral lidocaine [30].

A meaningful limitation of this study pertains to the underlying nature of the comparison presented herein. Specifically, we compared two OVDs with different physicochemical and rheological properties, one with and the other without lidocaine. Therefore, we have reached conclusions encompassing potential confounders i.e., between the presence of lidocaine in Visthesia and the different properties of the two OVDs. In addition, the possibility of recall bias interfering with intraocular pain may not be ruled out, but has essentially been minimized as patients were asked to rate their intraoperative pain immediately after surgery.

## Conclusions

In conclusion, our study suggests that Viscoat is more safe and protective for the corneal endothelium during uneventful phacoemulsification cataract surgery, while Visthesia is in superior position regarding intraoperative pain. Patients of both groups acquired excellent visual acuity postoperative, pointing on the value of cataract surgery when necessary. Finally, this is the first study comparing OVDs in terms of macular thickness, finding that Visthesia cause a greater increase in macular thickness postoperatively than Viscoat, although it reaches normal ranges in both groups.

## Competing interests

The authors declare that they have no competing interests.

## Authors' contributions

MM conceived of the study, participated in its design, collected data and revised critically the manuscript. IP participated in the design of the study, collected data and drafted the manuscript. TS participated in the design of the study, performed the statistical analysis and revised critically the manuscript. All authors read and approved the final manuscript.

## Pre-publication history

The pre-publication history for this paper can be accessed here:

http://www.biomedcentral.com/1471-2415/11/9/prepub
